# Elevated depression and anxiety symptoms among younger adults in Germany during the post-pandemic period

**DOI:** 10.1186/s12889-026-27600-0

**Published:** 2026-04-30

**Authors:** Peter Lena, Sitarski Emily, Hammann Jasmin, Köhler-Dauner Franziska, Fegert Jörg M., Fegert Jonas

**Affiliations:** 1 Department of Child and Adolescent Psychiatry, Psychosomatics and Psychotherapy, University Hospital of Ulm, Steinhövelstraße 5, 89075 Ulm, Germany; 2 German Center for Mental Health (DZPG), Partner Site Mannheim-Heidelberg-Ulm, Mannheim, Germany; 3 FZI Research Center for Information Technology, Karlsruhe, Germany; 4 KIT Karlsruhe Institute of Technology, Digital Democracy & Participation Research Group, Karlsruhe, Germany; 5 Competence Center Public, Child Mental Health, Partner Site Ulm, Steinhövelstraße 5, 89075 Ulm, Germany

**Keywords:** Generational differences, Generation Z, Mental health, Depression, Anxiety, Prevention

## Abstract

**Background:**

Adolescence and early adulthood are crucial periods marked by identity formation and increased vulnerability to mental health issues, with most mental disorders beginning before the age of 25. Today’s youth face amplified risks during a period characterized by multiple societal crises like the COVID-19 pandemic, climate change and economic instability, potentially disrupting age-typical developmental tasks and contributing to rising rates of anxiety and depression. This study aimed to investigate longitudinal patterns of anxiety and depression symptoms across four generational cohorts.

**Methods:**

This study drew on data from the German subsample of the ongoing “SOSEC – Social Sentiment in Times of Crises” project. In total, 25,143 individuals contributed 112,858 survey entries, responding to items on depression and anxiety symptoms based on modified versions of the PHQ-9 and GAD-2. Generational groups were defined based on year of birth (Generation Z: 1995–2007; Generation Y: 1980–1994; Generation X: 1965–1979; Baby Boomers/Traditionalists: 1925–1964). Linear mixed-effects models were used to examine associations of generation, survey period, gender and employment status with reported symptom levels, including interaction terms and post-hoc comparisons.

**Results:**

On average, Generation Z reported higher levels of depression and anxiety symptoms compared to older generations across all survey periods (all *p* < .001). Women reported higher symptom levels than men and employment was associated to lower symptom levels.

**Conclusions:**

The findings indicate persistently elevated levels of self-reported depression and anxiety symptoms among younger respondents, particularly Generation Z. Associations with employment suggest potential avenues for targeted prevention. Cautious interpretation is warranted, given the observational design and potential confounding factors. These results highlight the importance of monitoring mental health trajectories in young populations during periods of societal stress.

**Supplementary Information:**

The online version contains supplementary material available at 10.1186/s12889-026-27600-0.

## Introduction

Adolescence and early adulthood are critical life phases in which individuals face a broad range of developmental tasks. In addition to achieving biological and sexual maturity, this period involves the consolidation of personal identity, the formation of intimate peer relationships and the gradual development of autonomy within a given socio-cultural context [1, 2]. These formative years also play a central role in shaping long-term health behaviors and attitudes, as values and patterns established in this stage often persist into adulthood [3].

From a clinical perspective, adolescence and early adulthood represent a period of heightened vulnerability for the onset of mental disorders. It is estimated that half of all diagnosable mental health disorders begin during adolescence and that up to three-quarters manifest by the age of 25 [4–6]. While multiple societal crises currently co-occur, it is difficult to directly compare their overall severity with those experienced by previous generations. However, younger cohorts may experience these stressors within a distinct informational environment characterized by continuous media exposure. In addition to the traditional developmental challenges, contemporary adolescents and young adults are confronted with what has been described as a “polycrisis” – a convergence of overlapping societal crises including the COVID-19 pandemic, climate change, geopolitical conflict, economic precarity and political instability [6, 7]. These cumulative stressors may be associated with changes in normative developmental patterns and with elevated symptoms of internalizing disorders such as depression and anxiety [8].

An increase in psychosocial disorders among young people has been documented since the mid-twentieth century [9] but the COVID-19 pandemic in particular has exemplified how such crises can disproportionately affect children, adolescents and young adults. National studies report rising rates of anxiety and depression symptoms during the pandemic, especially among younger individuals and women [10, 11]. Findings across Europe similarly show a significant increase in symptoms of depression and anxiety among children and adolescents during this period [12, 13]. Interestingly, symptom levels ofdepression and anxiety were observed to be significantly higher, when the restrictions were more stringent or school-closures occurred [12, 13]. Limited contact with peers, financial strain and returning to the parental home were identified as further risk factors for the decline of life satisfaction and mental health over the COVID-19 pandemic among young people [14].

From a developmental perspective, adolescence is a transitional period in which peer relationships are central for identity formation, autonomy, and social development. Consequently, pandemic-related school closures and contact restrictions likely disrupted age-typical developmental tasks and peer-related developmental needs. Empirical studies conducted during the pandemic have shown that reduced school connectedness and increased social isolation were associated with higher levels of anxiety and depression symptoms among adolescents [15]. In addition, unmet developmental tasks have been linked to increased psychological burden in adolescents with mental disorders [16].

Regarding mental health service use, existing literature suggests that stigma and structural access barriers may still hinder help-seeking among adolescents and young adults, even in cohorts that are generally more open to discussing mental health. It is therefore more appropriate to describe persistent barriers to service use rather than assuming uniformly higher stigma in Generation Z [17].

Generational comparisons further highlight the burden on younger cohorts. The 2022 McKinsey Health Survey shows that Generation Z reports significantly poorer mental health than Generations Y, X and the Baby Boomer cohort [18]. While some studies suggest that younger individuals report higher levels of psychological distress, evidence regarding differences in mental health service use or stigma across generations remains mixed and requires further investigation. These findings are relevant given the well-documented long-term implications of early-onset mental health problems, which can affect educational achievement, social integration, and general health outcomes over the life course [19, 20].

Moreover, the public discourse surrounding young people’s mental health is often laden with stigma and generational stereotypes. Some narratives portray Generation Z as particularly vulnerable, though empirical support for such claims is limited. These narratives may influence help-seeking behaviors and open discussion and reinforce self-stigmatization in young people. Stigma has been implicated in worsening mental health temporal patterns and treatment outcomes [21, 22] as well as being a significant barrier to seeking treatment [23], reflecting the broader social and cultural barriers that influence psychological well-being.

These findings underscore the importance of empirically grounded, generation-sensitive research to better understand how different age cohorts experience, respond to, and cope with societal crises. From a lifespan developmental perspective, differences in mental health across age groups may also reflect life-stage related processes rather than cohort-specific characteristics. Older adulthood is often associated with increased emotional regulation, greater psychological stability, and accumulated coping resources, which may buffer the psychological impact of societal stressors. In contrast, adolescence and emerging adulthood represent periods of heightened sensitivity to environmental stressors due to ongoing identity formation, social role transitions, and economic uncertainty. Thus, differences observed between generational cohorts in cross-sectional comparisons may reflect both developmental stage and cohort-specific experiences, making it important to interpret these patterns cautiously. At the same time, younger cohorts may experience contemporary crises within a distinct informational environment. In particular, Generation Z has grown up with pervasive digital connectivity and constant exposure to global events through social media platforms. This continuous flow of information, often accompanied by emotionally salient imagery and rapid news cycles, may intensify perceived threat and psychological distress compared to previous generations who encountered such events through more limited media channels. Consequently, both developmental stage and changes in the media landscape may shape how different age groups experience and respond to ongoing societal crises.

Since cross-sectional studies typically offer only a momentary snapshot, our analyses aimed to observe whether previously reported findings on this topic are reflected in our data. This study investigated the temporal patterns of depression and anxiety symptoms over a period of more than two and a half years in a large, representative German sample encompassing four generational cohorts. More specifically, it examined how these symptoms evolve during times of societal crisis and whether younger age groups, particularly individuals belonging to Generation Z, report higher levels of depression and anxiety symptoms compared to older cohorts. Based on previous evidence, we expected that younger cohorts, particularly Generation Z, would report higher levels of depression and anxiety symptoms compared to older cohorts and that these differences would persist across survey periods.

## Methods

### Design

This research was carried out as part of the “SOSEC – Social Sentiment in Times of Crises” project, a representative panel survey conducted in both Germany and the United States. The SOSEC panel is implemented through an online survey platform operated by the research partner FZI Research Center for Information Technology in collaboration with the panel provider CINT. Participants are recruited from an online access panel using quota sampling to approximate national distributions for key demographic variables such as age, gender, and region. While this approach improves demographic representativeness, it does not constitute a true random sample. Respondents receive invitations through a mobile application and can participate in surveys at weekly to bi-weekly intervals. Participants receive small incentives for each completed survey. Data were collected between November 2022 and April 2025. The project aims to examine and compare trends in social sentiment across the two countries. To this end, roughly 1,500 participants in Germany and 3,000 participants in the United States take part in an online survey on a weekly to bi-weekly basis, which started in November 2022 and is still ongoing.

### Participants

The survey commenced in November 2022, with respondents receiving the same set of questions through an app at weekly to bi-weekly intervals. By April 2025, a total of 75 survey waves had been completed, yielding 122,120 survey entries from 26,237 participants. Each survey wave included approximately 1,500 respondents in Germany. Participation was not restricted to a fixed cohort; rather, individuals could participate in multiple survey waves while new respondents were continuously recruited by the panel provider. Consequently, the cumulative number of unique participants across all survey waves exceeded the number of respondents per wave, resulting in a total of 26,237 individuals contributing data to the study. Participants who had not completed the task on the day of the survey were excluded. Participants under 18 years of age were excluded to ensure comparability across generational groups and because the survey instruments were designed for adult respondents. The final dataset, containing complete information on the variables of interest, included 25,143 participants and 112,858 survey entries. Participation across survey waves varied considerably. While some respondents participated only once, others contributed data repeatedly. On average, participants completed 4.49 survey waves (SD = 7.98), with a range from 1 to 64 observations per individual.

### Measures

Depression symptoms were measured with two adapted items out of the PHQ-9 [24] that were rated on a 7-point Likert scale ranging from 1 (fully agree) to 7 (disagree at all). The two items were “I have little interest or pleasure in doing things.” and “I feel down, depressed or hopeless.”. Anxiety symptoms were measured with the adapted items out of the GAD-2 [25] “I feel not able to stop or control worrying.” and “I feel nervous, anxious or on edge.” that were rated on that same 7-point Likert scale. The two-item scale measuring depression symptoms achieved a Spearman-Brown coefficient of 0.879 and the one measuring anxiety symptoms achieved a coefficient of 0.796, both indicating acceptable internal consistency. For further calculation, the items were reversed, and the mean value was calculated, so that higher values indicate stronger endorsement of symptoms. The use of these adapted items provides brief self-report indicators of symptom endorsement rather than diagnostic measures. Therefore, caution is warranted when interpreting symptom levels in terms of clinical severity or prevalence.

Generational groups were determined based on participants’ reported year of birth. Following Schnetzer [26], the cohorts were defined as Generation Z (1995–2007), Generation Y (1980–1994), Generation X (1965–1979), and Baby Boomers/Traditionalists (1925–1964). For the purposes of this study, the Traditionalist and Baby Boomer cohorts were merged to simplify the analysis and presentation of generational differences. Since the focus is on Generation Z, separating the two oldest groups was deemed unnecessary for addressing the research questions. It should be noted that the use of generational categories in this study serves as an analytical grouping based on birth year. Given the observational design and the concurrent measurement of different age groups, the study cannot disentangle age, period, and cohort effects. Therefore, generational comparisons should be interpreted cautiously and may partly reflect life-stage differences rather than purely cohort-specific characteristics. It should be noted that these adapted items represent brief self-report indicators of symptom endorsement rather than clinically validated diagnostic measures. The use of a 7-point agreement scale differs from the original PHQ-9 and GAD-2 scoring, and therefore the resulting values cannot be directly interpreted in terms of clinical severity or prevalence.

### Analysis

Statistical analyses were conducted using R version 4.4.3, with a significance level of α = 0.05 applied throughout. The study hypothesis was evaluated using linear mixed-effects models via the *lmerTest* package [27]. The primary hypothesis concerned differences in symptom levels between generational groups and their variation across survey periods. Given that only 40.5% of participants completed the survey more than once, random slopes could not be reliably estimated and models therefore assumed a fixed slope. To capture medium-term trends and minimize short-term variability, data were aggregated into three-month intervals, resulting in ten survey periods for the analysis. This aggregation also simplified the interpretation of generational temporal patterns and reduced model complexity. For sensitivity purposes, the data were additionally aggregated into two- and four-month intervals. As presented in Additional file 1, the overall patterns of results remained highly consistent, supporting the robustness of the three-month aggregation. Main effects and interaction terms were examined using analysis of variance with type III sums of squares, implemented via the anova() function from the *car* package [28]. The mean values of depression and anxiety symptoms served as dependent variables, while generation, survey period, gender, employment status and the interaction between generation and survey period were included as predictors. Post-hoc comparisons were performed using the *emmeans* package [29] to identify specific group differences. To examine potential attrition bias, additional analyses were conducted to test whether baseline depression and anxiety symptom levels predicted subsequent participation in later survey waves. These analyses were performed using logistic regression models. In addition, sensitivity analyses were conducted including age as a continuous predictor to examine whether the observed differences between generational groups were robust to modelling age directly. Results of these analyses are reported in Additional file 1. As an additional robustness check, supplementary analyses were conducted restricting the sample to participants with repeated observations (minimum of 3 or 5 survey waves). These analyses yielded comparable patterns of results, indicating that the main findings were robust across participants with varying numbers of observations (see Additional file 1). Given the large sample size, statistical significance should be interpreted with caution, and effect sizes are reported to aid interpretation of the magnitude of differences.

## Results

### Descriptive statistics

Descriptive statistics for the study sample by generation are presented in Table 1. The largest proportion (36.7%) belonged to Generation Z, followed by 24.6% from Generation Y, 19.2% from Generation X, and 19.4% from the combined Baby Boomer/Traditionalist group. A majority of respondents were female (55.0%). Across survey entries, most participants reported being employed (63.4%). The mean value for depression symptoms was 3.08 (SD = 1.64) and for anxiety symptoms it was 3.39 (SD = 1.60).

**Table 1:** Sample characteristics by generation

	Generation			
	Z	Y	X	TB
Observations	17809	23224	28627	42198
Participants	9240	6193	4834	3876
Participation				
M	1.93	3.75	5.92	8.86
SD	2.91	6.47	9.30	11.81
% Female	63.9	47.1	47.1	44.2
% Employed	91.9	88.5	79.8	27.3
Depressive symptoms				
M	3.70	3.38	3.09	2.65
SD	1.50	1.65	1.70	1.53
Anxiety symptoms				
M	4.14	3.68	3.37	2.94
SD	1.39	1.56	1.65	1.53

### Linear mixed-effects models

Linear mixed-effects models were employed to evaluate the proposed hypothese. Initially, the intraclass correlation was calculated using an intercept-only model for each outcome. For depression symptoms, the intercept-only model produced an intraclass correlation of 0.806, indicating that 80.6% of the total variance is due to differences between individuals. Anxiety symptoms showed a comparable pattern, with an intraclass correlation of 0.745. These results justify the use of linear mixed-effects models to account for the nested structure of repeated surveys within individuals.

Analysis of depression symptoms revealed significant main effects for generation (χ^2^ = 808.06, *p* < 0.001), survey period (χ^2^ = 103.75, *p* < 0.001), gender (χ^2^ = 71.13, *p* < 001), and employment status (χ^2^ = 176.83, *p* < 0.001). Additionally, a significant interaction between generation and survey period emerged (χ^2^ = 89.93, *p* < 0.001), indicating that depression symptom temporal patterns over time varied across generations. Female participants exhibited significantly higher depression symptom levels than males (*b* = 0.16, SE = 0.02, *t* = 8.43, *p* < 0.001), whereas being employed was associated with lower levels of depression symptoms (*b* = − 0.26, SE = 0.02, *t* = − 13.30, *p* < 0.001), controlling for all other predictors in the model.

Post-hoc comparisons demonstrated that Generation Z reported higher depression symptoms than all older generations across all survey periods (all *p* < 0.001). Differences between Generation Z and the Baby Boomer/Traditionalist cohort ranged from 1.055 to 1.408 scale points. Comparisons with Generation X yielded slightly smaller differences, ranging from 0.501 to 0.765 points, while differences with Generation Y were the smallest but still significant, ranging from 0.192 to 0.376 points. A full summary of the depression symptom results is presented in Additional file 2. To aid interpretation, standardized effect sizes (Cohen’s d) were calculated based on residual standard deviations from the linear fixed-effects models. Cohen’s d ranged from 0.46 to 1.70 reflecting small to large differences after accounting for survey period, gender and employment status.

**Fig. 1:**
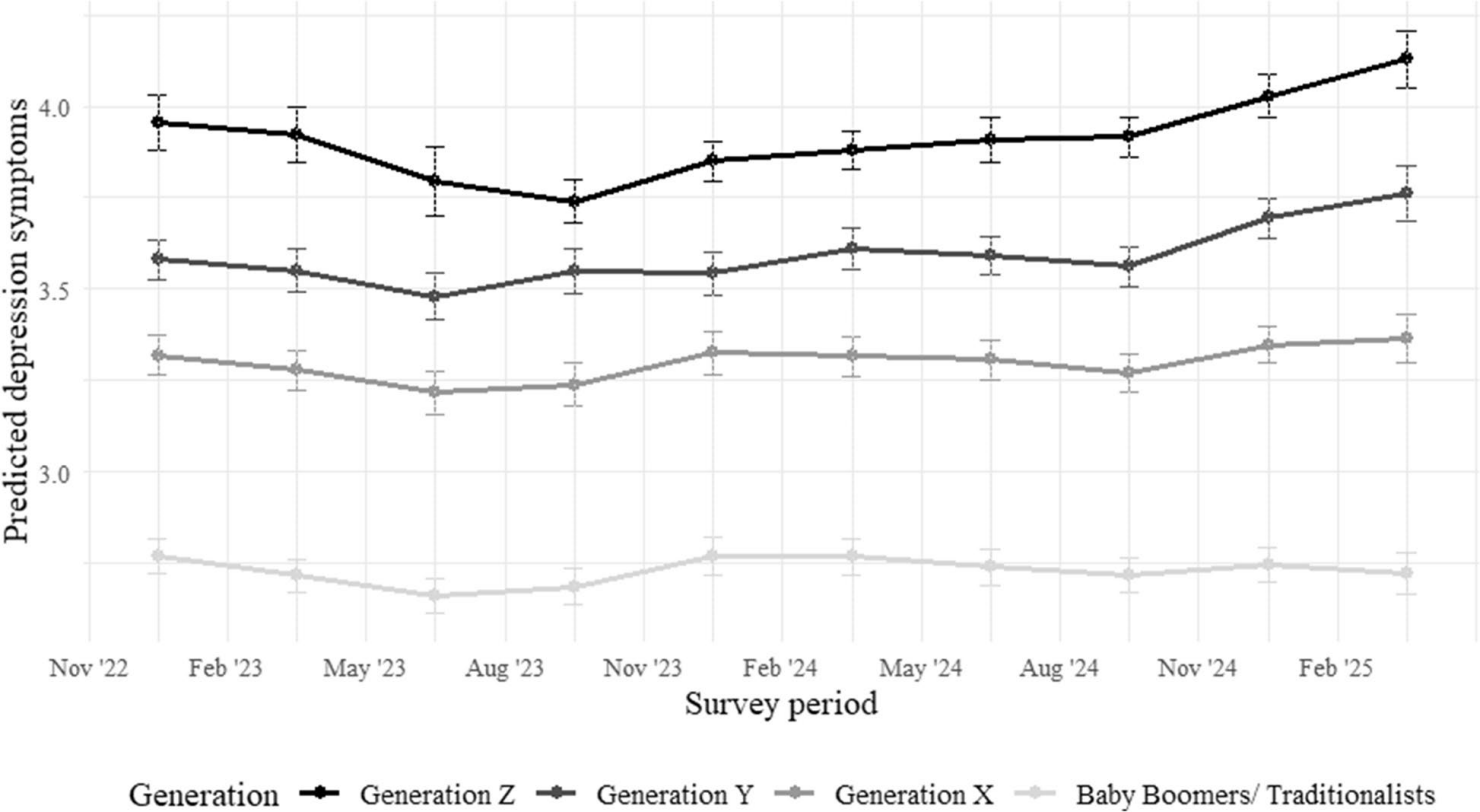
Estimated marginal means and 95% CI of depression symptoms across all generations and survey periods. *Notes.* Values are based on fixed effects estimates from the linear mixed-effects model

In addition to generational differences, descriptive trends across the ten survey periods were examined. Among Generation Z, the depression symptom levels slightly decreased between late 2022 and summer 2023, followed by a continuous increase through early 2025. Similar but less pronounced patterns were observed in Generations Y and X. Depression symptoms in Baby Boomers/Traditionalists remained comparatively stable and consistently lower across all time points. Figure 1 displays the estimated means and 95% confidence intervals over time.

A comparable pattern emerged for anxiety symptoms. Significant main effects were observed for generation (χ^2^ = 745.30, *p* < 0.001), survey period (χ^2^ = 40.66, *p* < 0.001), gender (χ^2^ = 327.69, *p* < 0.001), and employment (χ^2^ = 130.46, *p* < 0.001), as well as a significant interaction between generation and survey period (χ^2^ = 86.57, *p* < 0.001).Females reported higher anxiety symptom levels than males (*b* = 0.32, SE = 0.02, *t* = 18.10, *p* < 0.001), while employment was associated with lower anxiety levels (*b* = − 0.22, SE = 0.02, *t* = − 11.42, *p* < 0.001), after accounting for other predictors.

Post-hoc contrasts revealed that Generation Z consistently exhibited higher anxiety symptoms than each of the older generations throughout all survey periods (all *p* < 0.001). Estimated differences between Generation Z and Baby Boomers/Traditionalists ranged from 1.158 to 1.436 points, while differences with Generation X varied between 0.638 and 0.775 points. The smallest but still significant differences were observed in comparison with Generation Y, ranging from 0.313 to 0.412 points. A complete overview of the anxiety symptom results can be found in Additional file 3. To aid interpretation, standardized effect sizes (Cohen’s d) were calculated based on the residual standard deviations from the linear mixed-effects models. Cohen’s d ranged from 0.49 to 1.59 reflecting small to large differences after accounting for survey period, gender and employment status.

**Fig. 2:**
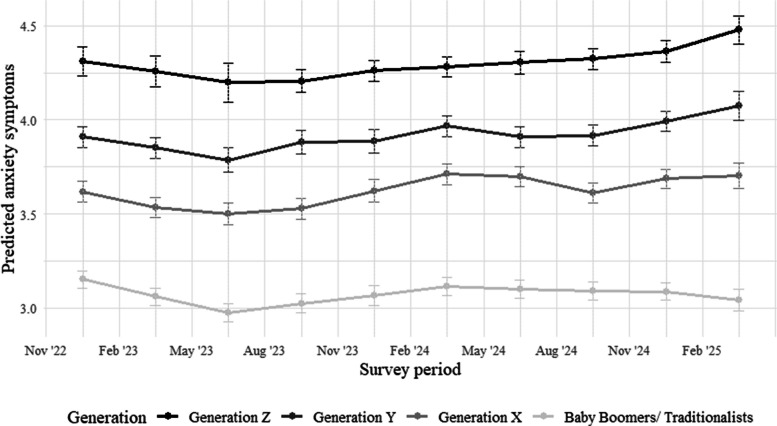
Estimated marginal means and 95% CI of anxiety symptoms across all generations and survey periods. *Notes.* Values are based on fixed effects estimates from the linear mixed-effects model

Across the ten survey periods, anxiety symptoms showed small temporal fluctuations across all generations. In Generation Z, estimated means declined slightly from late 2022 to mid 2023, followed by a gradual and continuous increase through early 2025. A similar but less pronounced pattern was observed for Generation Y, with a mid period dip and subsequent rise toward the end of the observation window. Generation X showed comparable fluctuations, with a slight decrease early on and a subsequent increase in later survey periods. The Baby Boomers/Traditionalists exhibited comparatively flatter temporal patterns, with only minimal variation over time and consistently lower symptom levels relative to the younger cohorts. These temporal trends are illustrated in Fig. 2, displaying the estimated means and 95% confidence intervals over time.

## Discussion

The aim of this study was to investigate the severity of symptoms of depression and anxiety in different generations and how these symptoms changed over a period of two and a half years. The results indicate that younger adults, particularly those in Generation Z, reported higher levels of selft-reported depression and anxiety symptoms across repeated cross-sectional observations within a panel design. Overall both, depression and anxiety symptoms, slightly increased over time among Generation Z, whereas for the Baby Boomers/Traditionalists, for example, depression symptoms returned to their initial level in the last survey period and anxiety symptoms even decreased overall in descriptive terms. Importantly, the present design does not allow causal conclusions regarding cohort-specific vulnerability, clinical disorder prevalence, or the underlying drivers of these differences.

These findings are broadly consistent with recent longitudinal and repeated cross-sectional studies which report elevated levels of psychological distress among younger adults during and after the COVID-19 pandemic. For example, several studies have reported persistent age gradients in depression and anxiety symptoms, with younger cohorts showing higher levels compared to older adults even after the acute phase of the pandemic [18]. Importantly, these findings should not be interpreted as indicating that mental health problems are inherent to a specific generation. Rather, the reported differences reflect self-reported symptom levels, which may be influenced by developmental stage, life circumstances, or contextual factors. Sensitivity analyses including age as a continuous predictor showed similar results, indicating that differences between generational groups likely reflect general patterns of higher symptom levels among younger adults rather than cohort-specific vulnerability. Older data on the prevalence of depression symptoms in Germany provide comparable results, indicating that the prevalence of depression symptoms is highest among 18- to 29-year-olds and decreases with increasing age [30]. More recent data from the RKI [31] suggest an increased prevalence of depression symptoms in younger cohorts compared to older cohorts, especially for women. Regarding anxiety symptoms, the health report by the RKI [32] has found that prevalence decreases with age, at least for women. Future research should examine whether cohort-by-gender interactions exist, as gender differences in mental health may vary across age groups due to differences in exposure to stressors and reporting patterns. Taken together, the results of the present study are consistent with previous findings of increased psychological distress among younger cohorts. At the same time, the observed differences between generational groups should be interpreted with caution. Because the study measures different age groups concurrently, it is not possible to disentangle age, period, and cohort effects. The higher symptom levels observed among Generation Z may therefore partly reflect well-established age gradients in internalizing symptoms rather than purely cohort-specific vulnerability. Longitudinal cohort designs would be required to separate these mechanisms more conclusively. Accordingly, the observed temporal patterns should be interpreted primarily as differences across survey periods rather than precise within-person changes over time.

From a developmental perspective, these differences may also reflect life-stage related processes. Adolescence and emerging adulthood are characterized by identity formation, social role transitions, and increased sensitivity to social and economic uncertainty, which may heighten vulnerability to psychological distress. In contrast, older adults often benefit from greater emotional regulation, accumulated coping resources, and more stable life circumstances, which may buffer the psychological impact of societal stressors. Consequently, differences observed between generational cohorts may partly reflect developmental stage rather than purely cohort-specific characteristics.

The descriptive reduction in both anxiety and depression symptoms from late 2022 to mid-2023across all generations could be related to the easing of pandemic-related restrictions and a broader stabilization of mental health reported in previous studies. However, the study did not directly measure these contextual factors, so this remains speculative. Similarly, the subsequent increase in symptoms, particularly among Generation Z, occurred during a period of accumulating socio-economic and geopolitical stressors, such as persistent inflation, geopolitical conflicts, and rising political polarization. These temporal patterns may reflect associations with broader contextual factors, but causal conclusions cannot be drawn.

Across models, employment was associated with lower self-reported depression and anxiety symptoms. This finding is consistent with previous research suggesting that employment may be linked to greater financial stability, daily structure, and social integration. However, this association should be interpreted with caution, as the relationship between employment and mental health is likely bidirectional and may be influenced by underlying factors such as age,socioeconomic status and baseline mental health.

These findings have implications for monitoring population mental health and for service planning, particularly with regard to younger age groups. They also highlight the need for future research designs that can more clearly disentangle age, period, and cohort effects, for example through longitudinal cohort studies or designs incorporating external contextual indicators.

### Limitations

Several methodological constraints should be considered. First, the longitudinal panel design entails conceptual complexities. Repeated assessments reflect not only cohort-related differences but also situational influences tied to specific economic, societal or social conditions. Therefore, observed temporal patterns may capture both relatively stable generational characteristics and situational fluctuations triggered by each survey. period In some cases, temporal patterns were similar across generations, emphasizing that interpretations regarding cohort-specific trends, particularly in relation to global events, remain hypothetical.

Participation was voluntary and incentivized, so self-selection processes cannot be ruled out. Of 25,143 participants, only 40.5% provided data on more than one occasion, with an average of 4.49 survey responses per participant. Consequently, analyses primarily capture population-level patterns rather than detailed within-person trajectories. Additional analyses restricted to participants with repeated observations (minimum of 3 or 5 survey waves) yielded comparable results, supporting the robustness of the main findings.

In addition, participation patterns differed by generation, with younger respondents contributing fewer repeated observations. While members of Generation Z contributed on average fewer than two survey waves (M = 1.93, SD = 2.91), participation was more sustained in older cohorts. Generation Y averaged 3.75 observations (SD = 6.47), Generation X 5.92 (SD = 9.30) and Baby Boomers/Traditionalists as many as 8.86 (SD = 11.80).

. Consequently, variations in sample composition across survey periods may partly influence the observed temporal patterns, reinforcing that findings primarily reflect population-level differences rather than individual trajectories.

Furthermore, the oldest cohort combined Baby Boomers and Traditionalists. While this simplified presentation, it also grouped individuals across a wide age range with potentially diverse life circumstances, which may have amplified contrasts with younger cohorts and should therefore be interpreted with caution. The consistency of results across alternative aggregation intervals (2-, 3-, and 4-month periods) suggests that the observed patterns are not driven by the specific choice of interval.

Depression and anxiety symptoms were assessed using two-item mean scores, which may not fully capture the multidimensional nature of these constructs and could limit construct validity [33]. Results are based solely on quantitative self-report data, which may be affected by socially desirable responding.

### Conclusion and future directions

This study provides evidence of generational differences in mental health, with Generation Z reporting consistently higher levels of depression and anxiety than older cohorts. These differences may reflect both the pressures associated with this life stage as well as the long-term consequences of the COVID-19 pandemic. Longitudinal, population-based research is essential not only for identifying risk and resilience factors within and between generational groups, but also for observing the impacts of concurrent societal events. Although such designs are influenced by situational fluctuations, they allow examination of how different generations respond over time and adapt to post-pandemic conditions.

Our results align with the Lancet Psychiatry Commission’s statement that the Youth Mental Health Crisis is ongoing and requires urgent action [34]. Social and political factors, including economic instability and limited support resources, may exacerbate stress among young people. While younger adults consistently report higher self-reported distress, the present data do not allow conclusions about the specific causes or the relative influence of societal versus generational factors.

Factors such as employment, which are associated with lower symptom levels, can inform preventive strategies. Supporting young people during key transitions, such as entering the workforce or starting higher education, may serve both as indicated prevention for those at heightened risk and as universal prevention to strengthen coping with age-typical developmental challenges. However, the relationship between employment and mental health is likely bidirectional and may be influenced by underlying factors such as age, socioeconomic status, and baseline mental health. Elevated symptom prevalence among young people reflects broader transitional challenges in times of crises, highlighting the need for targeted interventions. Continuous monitoring of mental health and societal sentiment, as implemented in SOSEC, can serve as an early warning system, guiding public health strategies and policy decisions to prevent further escalation of psychological strain among young people.

## Supplementary Information

Additional file1: Table 1a. – Sensitivity analysis of the survey period for depression symptoms. Sensitivity analysis examining whether the results of linear mixed-effects models, with depression symptoms as the dependent variable, differ when the survey period is divided into two-, three-, or four-month intervals. Table 1b – Sensitivity analysis of the survey period for anxiety symptoms. Sensitivity analysis examining whether the results of linear mixed-effects models, with anxiety symptoms as the dependent variable, differ when the survey period is divided into two-, three-, or four-month intervals. Table 2 – Baseline predictors of dropout. Logistic regression predicting participation in later survey waves based on baseline depression and anxiety symptoms, gender, and employment status. Odds ratios (OR) with 95% confidence intervals are reported. Table 3 – Robustness analysis for inclusion of age as continuous predictor. Linear mixed-effects models including age as a continuous predictor (centered, with quadratic term) to examine whether generational differences in symptom levels are robust. Table 4a – Robustness analyses for participants with repeated observations for depression symptoms. Linear mixed-effects models restricted to participants with at least 3 or 5 survey waves to examine robustness of generational differences in depression symptom levels. Table 4b – Robustness analyses for participants with repeated observations for anxiety symptoms. Linear mixed-effects models restricted to participants with at least 3 or 5 survey waves to examine robustness of generational differences in anxiety symptom levels.

Additional file 2: Table 5. Post-hoc contrasts for depression symptoms. Contains post-hoc contrasts for depression symptoms, showing pairwise comparisons between generational groups across all survey periods, with Generation Z as the reference group.

Additional file 3: Table 6. Post-hoc contrasts for anxiety symptoms. Contains post-hoc contrasts for anxiety symptoms, showing pairwise comparisons between generational groups across all survey periods, with Generation Z as the reference group.

## Data Availability

The data that support the findings of this study are available from FZI Research Center for Information Technology, but restrictions apply to the availability of these data, which were used under license for the current study, and so are not publicly available. Data are, however, available from the authors upon reasonable request and with permission of FZI Research Center for Information Technology.
