# Supplementary material for: Elevated depression and anxiety symptoms among younger adults in Germany during the post-pandemic period
Source: BMC Public Health. 2026 Apr 30;26:1420. doi: 10.1186/s12889-026-27600-0 (PMC13130811; doi:10.1186/s12889-026-27600-0)
Supplement: Supplementary file 1 — Additional file1: Table 1a. – Sensitivity analysis of the survey period for depression symptoms. Sensitivity analysis examining whether the results of linear mixed-effects models, with depression symptoms as the dependent variable, differ when the survey period is divided into two-, three-, or four-month intervals. Table 1b – Sensitivity analysis of the survey period for anxiety symptoms. Sensitivity analysis examining whether the results of linear mixed-effects models, with anxiety symptoms as the dependent variable, differ when the survey period is divided into two-, three-, or four-month intervals. Table 2 – Baseline predictors of dropout. Logistic regression predicting participation in later survey waves based on baseline depression and anxiety symptoms, gender, and employment status. Odds ratios (OR) with 95% confidence intervals are reported. Table 3 – Robustness analysis for inclusion of age as continuous predictor. Linear mixed-effects models including age as a continuous predictor (centered, with quadratic term) to examine whether generational differences in symptom levels are robust. Table 4a – Robustness analyses for participants with repeated observations for depression symptoms. Linear mixed-effects models restricted to participants with at least 3 or 5 survey waves to examine robustness of generational differences in depression symptom levels. Table 4b – Robustness analyses for participants with repeated observations for anxiety symptoms. Linear mixed-effects models restricted to participants with at least 3 or 5 survey waves to examine robustness of generational differences in anxiety symptom levels. [file 12889_2026_27600_MOESM1_ESM.pdf]

Table 1a

Sensitivity analysis of the survey period for depression symptoms

	2 months		3 months		4 months	
	χ^2	*p*	χ^2	*p*	χ^2	*p*
Generation	713.99	< .001	808.06	< .001	864.46	< .001
Survey period	127.99	< .001	103.75	< .001	102.67	< .001
Gender	70.12	< .001	71.13	< .001	70.76	< .001
Employment	176.87	< .001	176.83	< .001	176.52	< .001
Generation * Survey period	114.85	< .001	89.93	< .001	86.82	< .001

Table 1b

Sensitivity analysis of the survey period for anxiety symptoms

	2 months		3 months		4 months	
	χ^2	*p*	χ^2	*p*	χ^2	*p*
Generation	636.34	< .001	745.30	< .001	808.76	< .001
Survey period	59.11	< .001	40.66	< .001	40.03	< .001
Gender	327.65	< .001	327.69	< .001	329.88	< .001
Employment	131.37	< .001	130.46	< .001	131.36	< .001
Generation * Survey period	125.57	< .001	86.57	< .001	94.87	< .001

Table 2

Baseline predictors of dropout

	OR	*95% CI*	*p*
Depression symptoms	0.86	[0.68, 1.09]	.199
Anxiety symptoms	0.98	[0.76, 1.26]	.847
Gender (female)	0.55	[0.34, 0.88]	.015
Employment	0.60	[0.35, 1.02]	.061

Notes. Logistic regression predicting participation in later survey waves. Odds ratios >1 indicate higher likelihood of continued participation.

Table 3

Robustness analysis for inclusion of age as continuous predictor

	Depression symptoms		Anxiety symptoms	
	χ^2	*p*	χ^2	*p*
Generation	17.67	< .001	10.07	< .001
Age (centered)	135.49	< .001	179.65	< .001
Age^2	19.02	< .001	13.48	< .001
Survey period	107.84	< .001	44.27	< .001
Gender	63.47	< .001	308.37	< .001
Employment	231.29	< .001	187.63	< .001
Generation * Survey period	74.55	< .001	75.32	< .001

Notes. Linear mixed-effects models including generation and age (centered, with quadratic term) as predictors to test robustness of generational differences in depression and anxiety symptoms.

Table 4a

Robustness analyses for participants with repeated observations for depression symptoms

	3+ waves		5+ waves	
	χ^2	p	χ^2	p
Generation	390.83	< .001	283.68	< .001
Survey period	49.42	< .001	41.24	< .001
Gender	37.25	< .001	43.47	< .001
Employment	43.26	< .001	28.43	< .001
Generation * Survey period	49.99	< .001	45.35	< .001

Notes. Linear mixed-effects models fit separately for participants with ≥3 waves and ≥5 waves.

Table 4b

Robustness analyses for participants with repeated observations for anxiety symptoms

	3+ waves		5+ waves	
	χ^2	p	χ^2	p
Generation	416.28	< .001	301.42	< .001
Survey period	14.10	< .001	16.75	< .001
Gender	126.28	< .001	112.32	< .001
Employment	33.92	< .001	29.37	< .001
Generation * Survey period	57.18	< .001	58.77	< .001

Notes. Linear mixed-effects models fit separately for participants with ≥3 waves and ≥5 waves.
