# Supplementary material for: Elevated depression and anxiety symptoms among younger adults in Germany during the post-pandemic period
Source: BMC Public Health. 2026 Apr 30;26:1420. doi: 10.1186/s12889-026-27600-0 (PMC13130811; doi:10.1186/s12889-026-27600-0)
Supplement: Supplementary file 2 — Additional file 2: Table 5. Post-hoc contrasts for depression symptoms. Contains post-hoc contrasts for depression symptoms, showing pairwise comparisons between generational groups across all survey periods, with Generation Z as the reference group. [file 12889_2026_27600_MOESM2_ESM.pdf]

Table 5

Post-hoc contrasts for depressive symptoms

Survey period	Generation Y		Generation X		Baby boomers/ Traditionalists	
	Estimate (95% CI)	*p*	Estimate (95% CI)	*p*	Estimate (95% CI)	*p*
1	0.376 (0.257– 0.495)	<.001	0.636 (0.517 – 0.755)	<.001	1.186 (1.068– 1.304)	<.001
2	0.372 (0.250 – 0.494)	<.001	0.646 (0.525 – 0.767)	<.001	1.209 (1.089 – 1.329)	<.001
3	0.315 (0.167 – 0.463)	<.001	0.579 (0.435 – 0.723)	<.001	1.134 (0.992 – 1.277)	<.001
4	0.192 (0.085 – 0.300)	<.001	0.501 (0.396 – 0.605)	<.001	1.055 (0.953 – 1.158)	<.001
5	0.308 (0.205 – 0.411)	<.001	0.526 (0.423 – 0.629)	<.001	1.084 (0.982 – 1.185)	<.001
6	0.268 (0.173 – 0.364)	<.001	0.563 (0.467 – 0.658)	<.001	1.111 (1.015 – 1.207)	<.001
7	0.317 (0.216 – 0.418)	<.001	0.602 (0.500 – 0.704)	<.001	1.169 (1.066– 1.273)	<.001
8	0.354 (0.257 – 0.451)	<.001	0.646 (0.549 – 0.744)	<.001	1.198 (1.099– 1.297)	<.001
9	0.335 (0.237 – 0.433)	<.001	0.682 (0.584 – 0.779)	<.001	1.283 (1.182 – 1.383)	<.001
10	0.369 (0.228 – 0.510)	<.001	0.765 (0.632 – 0.898)	<.001	1.408 (1.278 – 1.538)	<.001

Notes. Generation Z serves as the reference group.
