# Supplementary material for: Elevated depression and anxiety symptoms among younger adults in Germany during the post-pandemic period
Source: BMC Public Health. 2026 Apr 30;26:1420. doi: 10.1186/s12889-026-27600-0 (PMC13130811; doi:10.1186/s12889-026-27600-0)
Supplement: Supplementary file 3 — Additional file 3: Table 6. Post-hoc contrasts for anxiety symptoms. Contains post-hoc contrasts for anxiety symptoms, showing pairwise comparisons between generational groups across all survey periods, with Generation Z as the reference group. [file 12889_2026_27600_MOESM3_ESM.pdf]

Table 6

Post-hoc contrasts for anxiety symptoms

Survey period	Generation Y		Generation X		Baby boomers/ Traditionalists	
	Estimate (95% CI)	*p*	Estimate (95% CI)	*p*	Estimate (95% CI)	*p*
1	0.401 (0.279 – 0.523)	<.001	0.691 (0.570 – 0.813)	<.001	1.158 (1.038– 1.278)	<.001
2	0.407 (0.280 – 0.533)	<.001	0.723 (0.598 – 0.847)	<.001	1.196 (1.073 – 1.319)	<.001
3	0.412 (0.255 – 0.569)	<.001	0.697 (0.545 – 0.849)	<.001	1.222 (1.072– 1.372)	<.001
4	0.325 (0.215 – 0.435)	<.001	0.678 (0.572 – 0.785)	<.001	1.180 (1.077– 1.284)	<.001
5	0.375 (0.269 – 0.481)	<.001	0.638 (0.533 – 0.743)	<.001	1.195 (1.093– 1.298)	<.001
6	0.313 (0.217 – 0.410)	<.001	0.570 (0.474 – 0.666)	<.001	1.167 (1.071– 1.263)	<.001
7	0.396 (0.294 – 0.499)	<.001	0.605 (0.501 – 0.708)	<.001	1.206 (1.102– 1.310)	<.001
8	0.406 (0.309 – 0.504)	<.001	0.712 (0.614 – 0.810)	<.001	1.231 (1.133– 1.330)	<.001
9	0.372 (0.273 – 0.470)	<.001	0.679 (0.582 – 0.776)	<.001	1.279 (1.179– 1.378)	<.001
10	0.401 (0.262 – 0.541)	<.001	0.775 (0.642 – 0.907)	<.001	1.436 (1.307– 1.565)	<.001

Notes. Generation Z serves as the reference group.
